# Cognitive composite score association with Alzheimer’s disease plaque and tangle pathology

**DOI:** 10.1186/s13195-018-0401-z

**Published:** 2018-09-11

**Authors:** Michael Malek-Ahmadi, Kewei Chen, Sylvia E. Perez, Anna He, Elliott J. Mufson

**Affiliations:** 1 Banner Alzheimer’s Institute, 901 E. Willetta St, Phoenix, AZ USA; 2 Department of Neurobiology and Neurology, Barrow Neurological Institute, 350 W. Thomas Rd, Phoenix, AZ 85013 USA

**Keywords:** Amyloid, Tau, Cognition, Prevention, Pre-clinical, Neuropathology

## Abstract

**Background:**

Cognitive composite scores are used as the primary outcome measures for Alzheimer’s disease (AD) prevention trials; however, the extent to which these composite measures correlate with AD pathology has not been fully investigated. Since many on-going AD prevention studies are testing therapies that target either amyloid or tau, we sought to establish an association between a cognitive composite score and the underlying pathology of AD.

**Methods:**

Data from 192 older deceased and autopsied persons from the Rush Religious Order Study were used in this study. All participants were classified at their initial evaluations with a clinical diagnosis of no cognitive impairment (NCI). Of these individuals, 105 remained NCI at the time of their death while the remaining 87 progressed to mild cognitive impairment (MCI) or AD. A cognitive composite score composed of eight cognitive tests was used as the outcome measure. Individuals were classified into groups based on Consortium to Establish a Registry for Alzheimer’s Disease (CERAD) neuropathological diagnosis and Braak stage.

**Results:**

The rate of annualized composite score decline was significantly greater for the high CERAD (*p* < 0.001, *d* = 0.56) and Braak (*p* < 0.001, *d* = 0.55) groups compared with the low CERAD and Braak groups, respectively. Mixed-model repeated measure (MMRM) analyses revealed a significantly greater difference in composite score change from baseline for the high CERAD group relative to the low CERAD group after 5 years (Δ = −2.74, 95% confidence interval (CI) −5.01 to −0.47; *p* = 0.02). A similar analysis between low and high Braak stage groups found no significant difference in change from baseline (Δ = −0.69, 95% CI −3.03 to 1.66; *p* = 0.56).

**Conclusions:**

These data provide evidence that decreased cognitive composite scores were significantly associated with increased AD pathology and provide support for the use of cognitive composite scores in AD prevention trials.

## Background

The recent initiation of secondary prevention trials for Alzheimer’s disease (AD) [1, 2] has resulted in a paradigm shift in the AD clinical research field, with the focus on identifying individuals who are at risk of developing AD by virtue of age, genetic risk factors, and/or the presence of AD pathology through neuroimaging technologies. Until more robust associations between clinical outcomes and neuroimaging/fluid-based biomarkers are established [3], the gold standard measure of efficacy in AD clinical prevention trials will continue to be the clinical evaluation of cognitive changes over time. However, the cognitive assessments in AD clinical trials are not sensitive enough to detect changes in less cognitively impaired individuals [4, 5], and therefore their use in prevention trials remains dubious. To identify the earliest clinically meaningful changes in cognition, the use of a composite score comprised of several different cognitive tests has been proposed [6]. Given that the incidence of mild cognitive impairment (MCI) can vary greatly [7, 8], AD prevention trials that use time-to-event as an outcome require extended observation periods to accurately assess disease progression. As a result, it is important that prevention trials use measures of cognition that can demonstrate meaningful treatment effects in the preclinical stages of dementia with reasonable trial durations.

It has been suggested that preclinical AD can be characterized by subtle cognitive changes that are detectable via sensitive neuropsychological tests and reflect the initial stages of the disease process [9, 10]. In addition, preclinical AD is also characterized by a lack of functional decline [11], which puts greater significance on the ability to demonstrate treatment efficacy through cognitive assessments in AD prevention trials. Therefore, emphasis has been directed towards the creation and validation of cognitive composite scores as primary efficacy measures in AD prevention trials to detect subtle cognitive changes between treatment and placebo groups.

Several different cognitive composite measures have been developed [12–16] which are sensitive to subtle cognitive decline during the preclinical phase of AD. These composite scores were developed in different populations using differing variables (e.g., APOE ɛ4 status, amyloid positivity, postmortem AD neuropathology) to differentiate preclinical AD from age-similar controls. Others have proposed the Catch-Cog, a composite score that combines information from performance-based cognitive assessments with informant-based functional assessments [17]. Furthermore, the European Prevention of Alzheimer’s Dementia (EPAD) group has implemented a comprehensive battery of cognitive tests that assess several cognitive domains [18]. Many of the tests in the EPAD battery are widely used verbal or paper and pencil tests, while others are novel computer-based tasks that are intended to measure cognitive changes associated with specific cortical regions known to be differentially affected by AD pathology [18]. Although there is evidence demonstrating that these composite scores have good sensitivity to clinical change [12–16], it is unclear whether these particular composite scores correlate with autopsy-confirmed AD pathology.

Other groups have shown that cognitive composite scores correlate well with AD pathology among individuals with no cognitive impairment (NCI) who had not progressed to MCI or AD at the time of death [19–21]. Boyle et al. [19] showed that higher levels of both amyloid and tau load were associated with longitudinal decreases in global cognition after adjusting for age, sex, and education. Riley et al. [20] revealed that NCI individuals with a NIA-Reagan diagnosis of intermediate or high likelihood of AD had significantly greater antemortem cognitive decline relative to those with the no or low likelihood diagnoses. Monsell et al. [21] reported that NCI individuals with high levels of AD pathology had significantly greater decline on a composite score of attention/working memory when compared with NCI individuals with no evidence of AD pathology. Together, these studies provide evidence that elderly people that died with an antemortem clinical diagnosis of NCI but who displayed extensive AD pathology on brain neuropathological evaluation postmortem show a significant decline on a composite memory score.

Although it appears that cognitive composite score changes correlate with the presence of AD-related pathology among aged individuals who are cognitively stable, whether similar associations exist in individuals that progress to MCI or AD remains to be investigated. Since AD prevention trials are likely to enroll individuals who progress to MCI during the course of the study, understanding the nature of cognitive trajectories among progressors and nonprogressors is needed. Therefore, the aim of this study was to define the relationship between longitudinal changes on a cognitive composite score and postmortem plaque and neurofibrillary tangle (NFT) pathology among NCI individuals who did and did not progress to MCI and AD.

## Methods

The data examined were derived from 192 older deceased and autopsied persons who were classified as NCI at their initial clinical evaluation. At the last testing within 12 months prior to death, 105 of these individuals remained NCI while the remaining 87 progressed to MCI (*n* = 40) or AD (*n* = 47) (Table 1). Among those who progressed to MCI, 13 were classified as amnestic and 27 were classified as nonamnestic. Previous work by our group has shown that plaque and tangle pathology does not differ significantly between amnestic and nonamnestic MCI subjects in this cohort [22]. These individuals were participants in the Rush Religious Order Study (RROS) [23, 24], had no coexisting clinical or neurological conditions judged to contribute to cognitive impairment at their last clinical evaluation [23, 24], agreed to annual clinical evaluations, and signed an informed consent and an Anatomic Gift Act donating their brains at the time of death. Data from these subjects have been used in numerous clinical pathological studies supported by our ongoing NIA program project grant entitled the “Neurobiology of Mild Cognitive Impairment in the Elderly” (PO1AG14449). At the time of these studies, individuals were chosen from all available RROS participants that came to autopsy during a rolling admission [23]. In addition, those taking anticholinesterases or medication for depression were also excluded. The Human Investigation Committee of Rush University Medical Center approved this study.

**Table 1:** Demographic, cognitive, and postmortem data by progression status

	Progressors	Nonprogressors	*p* value	Effect size
*n*	87	105	NA	NA
Gender (male/female), *n*	27/60	54/51	< 0.001	NA
APOE e4 (carrier/noncarrier), *n*	25/62	18/87	0.06	NA
Age at baseline (years)	78.95 ± 5.93	76.40 ± 6.16	< 0.001	0.42
Age at death (years)	87.84 ± 5.67	84.48 ± 5.61	< 0.001	0.60
Education (years)	18.31 ± 3.22	18.20 ± 3.57	0.82	0.03
MMSE at baseline	28.28 ± 1.78	28.68 ± 1.39	0.08	0.25
MMSE proximate to autopsy	21.92 ± 6.61	28.30 ± 1.29	< 0.001	1.34
Length of follow-up (years)	7.65 ± 3.72	7.23 ± 4.75	0.50	0.10
Baseline composite score	67.24 ± 6.85	69.08 ± 7.37	0.08	0.26
Interval between last clinic visit and autopsy (months)	0.69 ± 0.55	0.68 ± 0.57	0.95	0.02
Postmortem interval (h)	6.95 ± 5.15	7.11 ± 6.95	0.85	0.03
Brain weight at autopsy (g)	1165.97 ± 131.11	1224.61 ± 142.93	< 0.001	0.43

Values are shown as mean ± standard deviation unless otherwise indicated

Effect size = Cohen’s *d*

*MMSE* Mini Mental State Examination, *NA* not applicable

### Clinical evaluation

Each of the participants underwent a uniform, structured, and clinical evaluation performed by a neurologist and a trained neuropsychological test technician [23, 25]. Medications used by the subjects within the previous 14 days of the examination were reviewed and classified. A neurologist reviewed the medical history, medication use, neurologic examination, results of cognitive performance testing, and the neuropsychologist’s opinion of cognitive impairment and dementia. Each participant was evaluated in their home, emphasizing findings deemed clinically relevant. Clinical diagnostic classification was performed as described previously [23, 25]. At the time of death, individuals with a clinical diagnosis of MCI or AD were classified as progressors and those classified as NCI were categorized as nonprogressors. Progression to MCI or AD was determined by performance on neuropsychological tests as well as a clinical examination by a neurologist. Based on these cognitive and clinical data, a diagnostic algorithm was used to determine the clinical status of each participant [26].

### Tissue preparation and neuropathological diagnosis

Brain accruement and processing was as described previously [25, 27]. Briefly, each brain was cut into 1-cm thick coronal slabs using a brain slice apparatus and hemisected. One hemisphere was immersion fixed in 4% paraformaldehyde (24–72 h) and cryoprotected (10% glycerol and 2% dimethyl sulfoxide in phosphate-buffered solution) until processing for immunohistochemistry.

Diagnostic blocks (mid-frontal, superior temporal, entorhinal cortex, hippocampus, inferior parietal cortex, basal ganglia, thalamus, and substantia nigra) from the opposite hemisphere were paraffin embedded and cut at 6 μm. Examination for cerebral infarctions was conducted as described previously [28]. Bielschowsky silver stain was used to visualize neuritic plaques (NPs), diffuse plaques (DPs), and NFTs. Sections were also immunostained for amyloid beta (Aβ) using antibody M0872 (1:100; Dako, CA) raised against Aβ_1–40_ and Aβ_1–42_. Paired helical filament tau (AT8; 1:800, Covance) immunohistochemistry was also used to label NFTs. Neuropathological diagnoses were determined according to Consortium to Establish a Registry for Alzheimer’s Disease (CERAD) [29] and Braak staging [30] as recommended by the NIA-Reagan criteria [31]. Exclusion criteria included mixed dementias, Parkinson’s disease, frontotemporal dementia, argyrophilic grain disease, vascular dementia, hippocampal sclerosis, stroke, and Lewy body disease. Lewy bodies in the substantia nigra, entorhinal, cingulate, midfrontal, middle temporal, and inferior parietal cortex were detected using α-synuclein immunohistochemistry as previously described [32] and scored semiquantitatively according to the severity and anatomical distribution, separating brainstem predominant, limbic/transitional, and diffuse neocortical types, depending on the anatomical distribution of the α-synuclein positivity [33, 34]. A board-certified neuropathologist or trained technician, blinded to clinical diagnosis, counted the number of NPs and DPs revealed by Bielschowsky silver stain and Tau immunohistochemistry using the phosphorylated paired helical filament tau AT8 marker for NFTs, respectively, in one square millimeter area (100× magnification) per cortical region as reported previously [35, 36]. NP and NFT counts used in this study were a summation of counts from the entorhinal cortex, CA1 hippocampus, midfrontal cortex, midtemporal cortex, and inferior parietal cortex.

### Cognitive composite score

The composite score was comprised of eight cognitive tests that included the CERAD Word List Delayed Recall, WMS-R Logical Memory (delayed recall), Category Fluency (Fruits and Animals), Symbol Digit Modalities Test, Ravens Progressive Matrices (9-item), Judgment of Line Orientation (15-item), MMSE Orientation to Time, and MMSE Orientation to Place. The composite score used in this study is based on that of Langbaum et al. [12], but was refined to reflect the selection of tests being used in on-going AD prevention trials [37, 38]. The tests that comprise this composite score are the same, or are analogous to, those used in other composite scores [12–14]. A list of the individual tests used to create the current composite score and others is shown in Table 2. The tests that form the composite score are intended to assess change in the cognitive domains of episodic memory (CERAD Word List Delayed Recall, WMS-R Logical Memory), attention/processing speed (Symbol Digit Modalities Test), executive function (Ravens Progressive Matrices), language (Category Fluency), visuospatial function (Judgment of Line Orientation), and orientation (MMSE Orientation to Time, and MMSE Orientation to Place). Several of these tests have been used in the formation of other composite scores [13, 14, 17]. Another score utilizes the Repeatable Battery for the Assessment of Neuropsychological Stats (RBANS), which includes List Learning and Story Memory tests to assess episodic memory as well as other tests that assess attention (Coding and Digit Span), language (Picture Naming and Semantic Fluency), and visuospatial function (Figure Copy and Line Orientation) [18].

**Table 2:** Composite score comparison between studies

Publication	Composite score components
Current study	CERAD Word List Delayed Recall, WMS-R Logical Memory (Delayed Recall), Category Fluency, Symbol Digit Modalities Test, Ravens Progressive Matrices (9-Item), Judgment of Line Orientation (15-Item), MMSE Orientation to Time, MMSE Orientation to Place.
Langbaum et al. [12]	East Boston Memory Test (Immediate Recall), WMS-R Logical Memory (Delayed Recall), Boston Naming Test (15-Item), Category Fluency, MMSE Orientation to Time, Ravens Progressive Matrices (9-Item), Symbol Digit Modalities Test
Donohue et al. [13]	Free and Cued Selective Reminding Test (Total Score), MMSE (Total Score), WMS-R Logical Memory (Delayed Recall), WAIS-R Digit Symbol Substitution
Coley et al. [14]	MMSE Orientation to Time, MMSE Orientation to Place, Free and Cued Selective Reminding Test (Total Score), Category Fluency, Trail-Making Test Part B
Jutten et al. [17]	ADAS-Cog Word Recall, ADAS-Cog Word Recognition, ADAS-Cog Orientation, Digit Span Backward, Controlled Oral Word Association Test, Category Fluency, Digit Symbol Substitution Test
Ritchie et al. [18]	RBANS List Learning, RBANS Story Memory, RBANS Figure Copy, RBANS Line Orientation, RBANS Picture Naming, RBANS Semantic Fluency, RBANS Coding, RBANS Digit Span, NIH Examiner Toolbox Dot Counting, NIH Examiner Toolbox Paired Associate Learning, NIH Examiner Toolbox Eriksen Flanker Task, Four Mountains Task

Individual raw scores for each test were standardized to a 0 to 1 scale by subtracting the minimum possible score for a test from the raw score and then dividing by the difference of the maximum and minimum possible scores:

$$ \mathrm{Standardized}\ \mathrm{score}=\left(\mathrm{raw}\ \mathrm{score}-\mathrm{minimum}\ \mathrm{possible}\ \mathrm{score}\right)/\left(\mathrm{maximum}\ \mathrm{possible}\ \mathrm{score}-\mathrm{minimum}\ \mathrm{possible}\ \mathrm{score}\right) $$

Since the Category Fluency test does not have an established maximum score, two standard deviations above the mean was used as the maximum. This method has been applied previously for a similar cognitive composite score [12]. No adjustments for directionality were needed since lower scores are indicative of decreased performance for all tests. The standardized scores for each test were then summed and divided by eight (the number of tests) to obtain an unweighted average. Finally, for scaling purposes, standardized scores were multiplied by 100.

An annualized rate of change for the composite score was calculated by subtracting the score at the last visit from the baseline score and then dividing by the difference in age between the two visits.

$$ \mathrm{Annualized}\ \mathrm{change}=\left(\mathrm{score}\ \mathrm{at}\ \mathrm{last}\ \mathrm{visit}-\mathrm{score}\ \mathrm{at}\ \mathrm{baseline}\right)/\left(\mathrm{age}\ \mathrm{at}\ \mathrm{last}\ \mathrm{visit}-\mathrm{age}\ \mathrm{at}\ \mathrm{baseline}\right) $$

### Statistical analysis

Between-group frequency differences for categorical variables were analyzed using the Chi-square test while between-group differences for continuous variables were compared with a two-sample *t* test. Annualized cognitive composite score change differences for CERAD neuropathological diagnosis and Braak stage were evaluated using a one-way analysis of variance (ANOVA). Braak stage was divided into three groups (0 to II, III, and IV to V) to maintain adequate group sizes for the ANOVA. This Braak stage grouping scheme also allowed for the transentorhinal stage (I and II) of NFT deposition to be differentiated from the intermediate limbic stage (III) [28]. Braak stage III was grouped independently since the transition from Braak stage III to IV is thought to coincide with the transition from normal cognition to dementia [39]. Stages IV and V were grouped together as none of the subjects were classified in stage VI. The Tukey HSD test was used for post-hoc group-wise comparisons.

Annualized composite score change was also analyzed when the cases were grouped based on pathology severity as measured by CERAD criteria and Braak stage. The low CERAD group consisted of those with the no AD classification, while possible, probable, and definite AD were classified as high CERAD. Low Braak stage consisted of individuals ranging from 0 to II and high Braak stage included individuals ranging from III to V.

In addition, mixed-model repeated measure (MMRM) analyses were used to examine change from baseline differences on the composite score between high and low pathology groups. In these analyses, time was treated as a categorical variable and data were restricted to the first six visits for each subject (baseline plus 5 years of follow-up). This follow-up length was selected to approximate the duration of current AD prevention trials [2, 37, 38]. Unstructured covariance structure was attempted for all models. In the event that the models did not converge, autoregressive order 1 (AR(1)) followed by variance component (VC) structures were used. Kenward-Roger approximation for degrees of freedom was used for all models. The MMRM models included fixed-effects for visit, age at baseline, gender, education, APOE ε4 carrier status, baseline composite score, pathology group, and visit by pathology group interaction. The primary outcome for each analysis was the least-squares difference of composite score change between the low and high pathology groups. Separate MMRM models were carried out to compare change from baseline differences in high versus low CERAD and high versus low Braak stage. A third MMRM model was carried out which grouped the participants based on both their CERAD and Braak stage status (high CERAD/high Braak, low CERAD/low Braak, and intermediate (high CERAD/low Braak and low CERAD/high Braak)).

The *t* tests and ANOVA were carried out using SYSTAT 13.1 (SYSTAT Software Inc., San Jose, CA). SAS Enterprise Guide 6.1 (SAS Institute, Cary, NC) was used for the MMRM analyses. Statistical significance was set at *p* ≤ 0.05.

## Results

### Demographic and postmortem characterization

Demographic, cognitive, and postmortem characteristics of the study sample are shown in Table 1. Females were more likely to progress to MCI/AD than males (*p* < 0.001). APOE ε4 status was not associated with disease progression (*p* = 0.06). Individuals who progressed to MCI or AD were approximately 2.5 years older than nonprogressors at baseline (*p* < 0.001). Progressors were also significantly older at their time of death relative to nonprogressors (*p* < 0.001); however, the two groups did not differ on years of education (*p* = 0.82), baseline Mini Mental State Examination (MMSE) (*p* = 0.08), length of follow-up (*p* = 0.50), or baseline composite score (*p* = 0.08). MMSE proximate to death was significantly lower for progressors (*p* < 0.001). The interval between the last clinic visit and death and postmortem interval were not significantly different (*p* = 0.95 and *p* = 0.85, respectively). Progressors had significantly lower brain weight at autopsy compared with nonprogressors (*p* < 0.001).

For the neuropathological variables (Table 3), CERAD neuropathological diagnosis prevalence of the no AD classification was significantly higher among nonprogressors (*p* < 0.001). Braak stage V was more prevalent among progressors than nonprogressors (*p* = 0.01).

**Table 3:** Neuropathological data by progression status

	Progressors (*n*)	Nonprogressors (*n*)	*p* value
CERAD neuropathological diagnosis			< 0.001
No AD	18	41	
Possible AD	6	14	
Probable AD	39	38	
Definite AD	24	12	
Braak stage			0.01
0	0	2	
I	9	19	
II	7	17	
III	24	31	
IV	23	31	
V	24	5	

*AD* Alzheimer’s disease

### Cognitive composite score and neuropathology associations

**Fig. 1:**
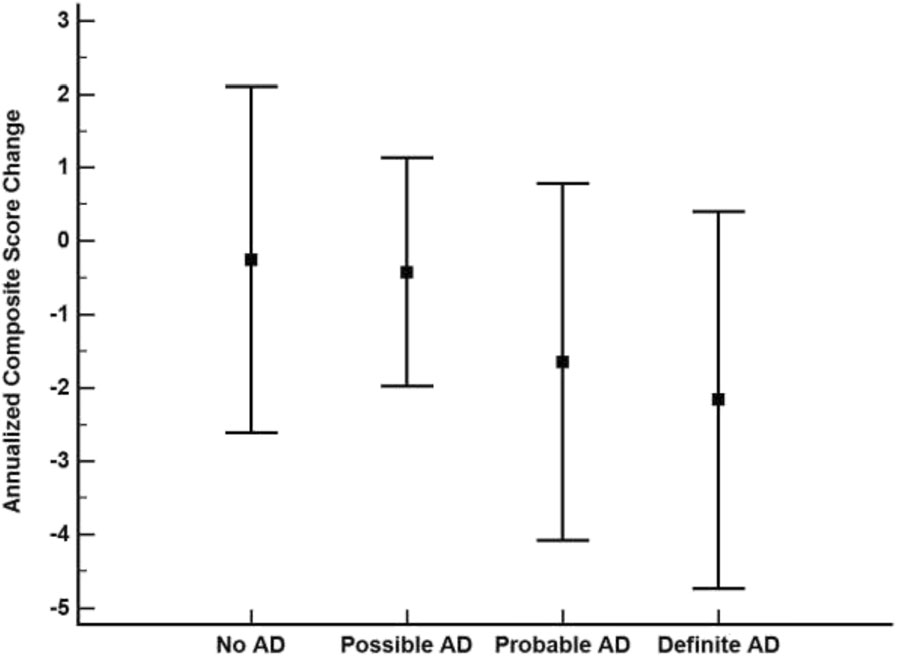
Annualized composite score change by CERAD neuropathological diagnosis. Boxes represent the mean and error bars are standard deviation. No Alzheimer’s disease (AD) vs probable AD, *p* = 0.01; no AD vs definite AD, *p* < 0.001; all other group-wise comparisons were not significantly different

**Fig. 2:**
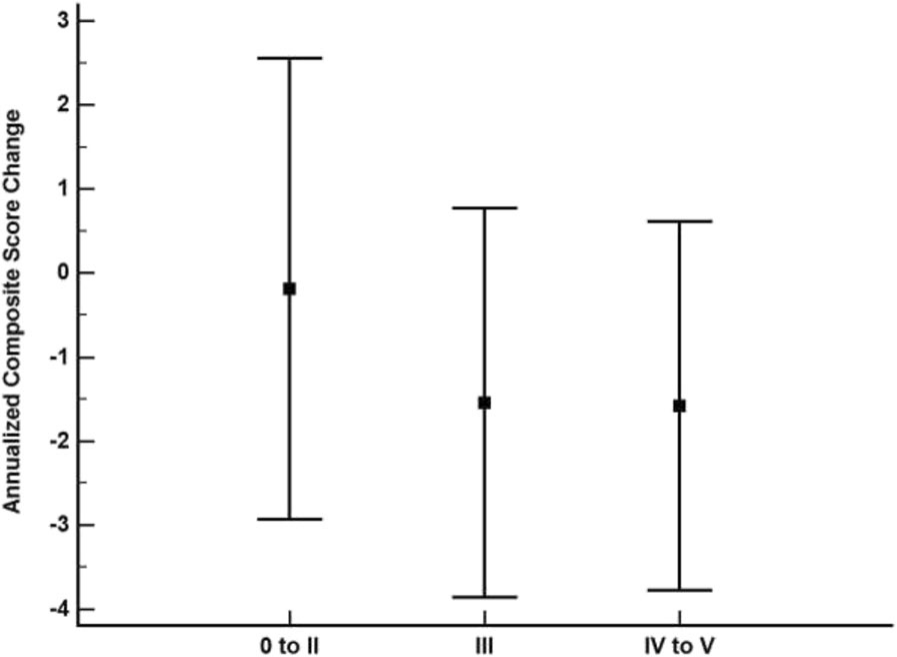
Annualized composite score change by Braak stage. Boxes represent the mean and error bars are standard deviation. 0 to II vs III, *p* = 0.01; 0 to II vs IV to V, *p* < 0.001; III vs IV to V, *p* = 0.99

Annualized composite score change between the CERAD classifications was significantly different (*p* < 0.001), with the no AD group showing a significantly slower rate of change relative to the probable AD (*p* = 0.01) and definite AD groups (*p* < 0.001) (Fig. 1). All other CERAD group-wise comparisons were not significantly different. For Braak stage, the 0 to II group showed a significantly slower annualized composite score change relative to the III (*p* = 0.01) and IV to V (*p* < 0.001) groups (Fig. 2). The Braak stage III and IV to V groups were not significantly different (*p* = 0.99).

Significant differences for the annualized cognitive composite score change was noted for both the CERAD and Braak stage groupings (both *p* < 0.001) with medium effect sizes (CERAD *d* = 0.56; Braak stage *d* = 0.55). Data for these analyses are shown in Table 4.

**Table 4:** Annualized cognitive composite score change differences for high and low pathology groupings

	High	Low	*p* value	Effect size
CERAD	−1.59 ± 2.40	−0.26 ± 2.36	< 0.001	0.56
	*n* = 133	*n* = 59		
Braak stage	−1.57 ± 2.23	−0.19 ± 2.75	< 0.001	0.55
	*n* = 138	*n* = 54		

Values are shown as mean ± standard deviation

Effect size = Cohen’s *d*

**Fig. 3:**
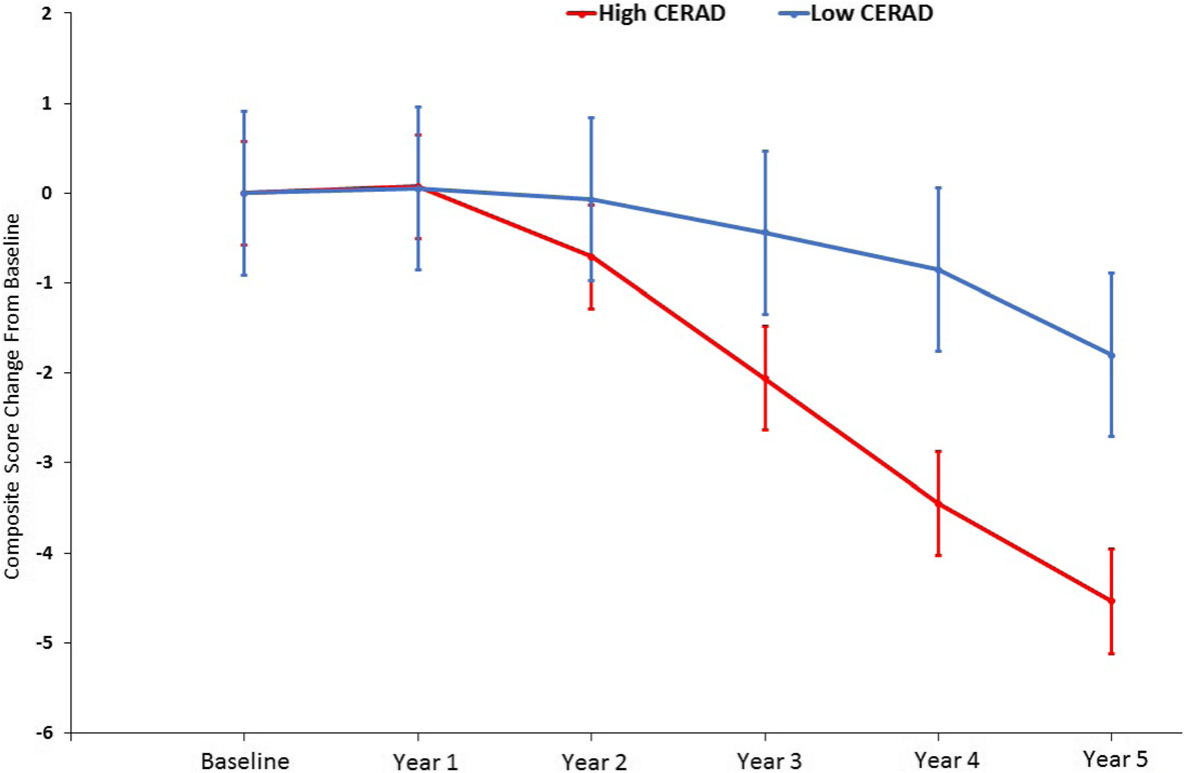
Least squares composite score estimates for high and low CERAD groups. Group difference in change from baseline was statistically significant (Δ = −2.74, 95% CI −5.01 to −0.47; *p* = 0.02). Error bars indicate standard error

**Fig. 4:**
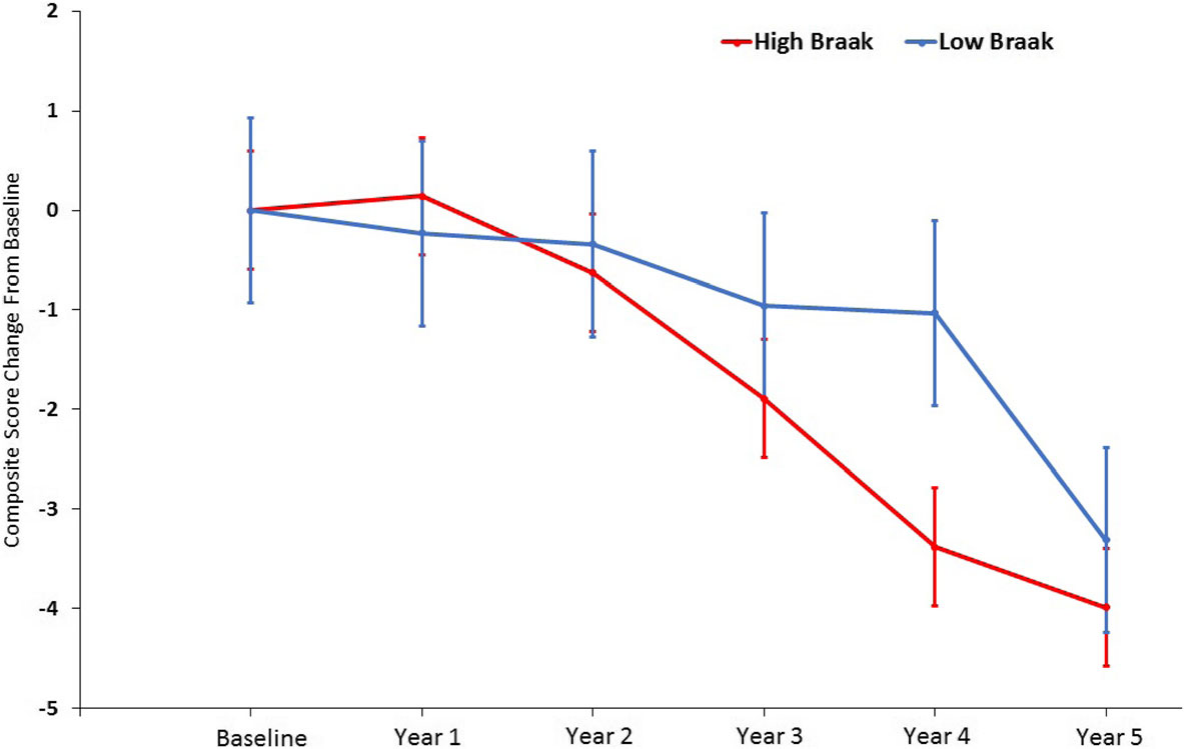
Least squares composite score estimates for high and low Braak stage. Group difference in change from baseline was not statistically significant (Δ = −0.69, 95% CI −3.03 to 1.66; *p* = 0.56). Within-group change from baseline was statistically significant for both groups (high Braak: Δ = −3.99, 95% CI −5.25 to −2.74; *p* < 0.001; low Braak: Δ = −3.30, 95% CI −5.38 to −1.23; *p* = 0.002). Error bars indicate standard error

The MMRM analysis for the CERAD grouping showed that the composite score change from baseline difference between the high and low CERAD groups was statistically significant (Δ = −2.74, 95% confidence interval (CI) −5.01 to −0.47; *p* = 0.02), with the high CERAD group showing a significantly greater change from baseline (Fig. 3). For Braak stage, the composite score change from baseline difference between the low and high groups was not statistically significant (Δ = −0.69, 95% CI −3.03 to 1.66; *p* = 0.56). Although the high/low Braak stage group difference was not statistically significant, independently, these groups both showed a statistically significant decline from baseline (high Braak: Δ = −3.99, 95% CI −5.25 to −2.74; *p* < 0.001; low Braak: Δ = −3.30, 95% CI −5.38 to −1.23; *p* = 0.002) (Fig. 4).

**Fig. 5:**
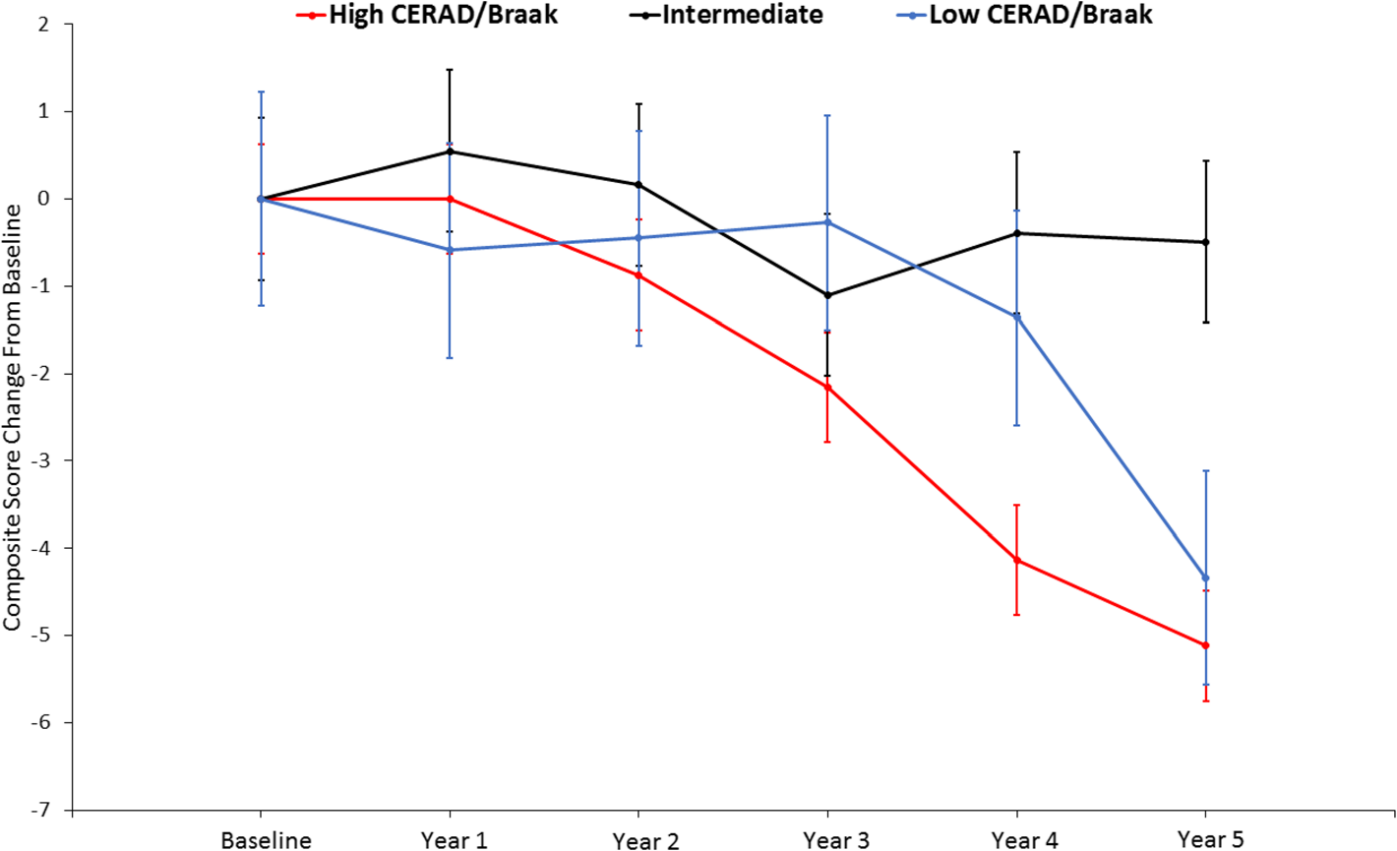
Least squares composite score estimates for high, intermediate, and low pathology groups. Group differences in change from baseline were statistically significant for intermediate vs. low (*p* = 0.01) and intermediate vs high (*p* < 0.001), but not for high vs. low (*p* = 0.61). Error bars indicate standard error

For the three-group analysis, the group sizes were as follows: high CERAD/high Braak, *n* = 56; intermediate, *n* = 53; low CERAD/low Braak, *n* = 83. The composite score change from baseline difference between the high CERAD/high Braak and the low CERAD/low Braak was not statistically significant (Δ = −0.78, 95% CI −3.81 to 2.24; *p* = 0.61). Further examination of the low CERAD/low Braak group found that eight individuals who progressed had composite score change from baseline estimates ranging from −2.15 to −21.70 (mean ± SD, −7.28 ± 6.61), which could explain the nonsignificant difference with the high CERAD/high Braak group. The intermediate group showed a significantly greater change from baseline than the low CERAD/low Braak group (Δ = 3.85, 95% CI 0.55–7.15; *p* = 0.02). Composite score decline for the high CERAD/high Braak group was significantly greater than that of the intermediate group (Δ = −4.63, 95% CI −7.09 to −1.58; *p* = 0.002) (Fig. 5). Within-group and between-group change from baseline estimates for each MMRM model are shown in Table 5. AR(1) covariance structure was used for all MMRM models due to a lack of convergence when using unstructured covariance.

**Table 5:** MMRM-estimated cognitive composite score change from baseline results

	Δ from baseline	*p* value	Δ from low group	*p* value
Low CERAD	− 1.80 (−3.79, 0.19)	0.08	NA	NA
High CERAD	−4.54 (−5.80, −3.28)	< 0.001	−2.74 (−5.01, − 0.47)	0.02
Low Braak	−3.30 (−5.38, −1.23)	0.002	NA	NA
High Braak	−3.99 (−5.25, −2.74)	< 0.001	−0.69 (−3.03, 1.66)	0.56
Low CERAD/low Braak	−4.34 (−7.09, −1.58)	0.002	NA	NA
Intermediate	−0.49 (−2.52, 1.54)	0.64	3.85 (0.55, 7.15)	0.02
High CERAD/high Braak	−5.12 (−6.48, −3.76)	< 0.001	−0.78 (−3.81, 2.24)	0.61

Values are shown as least-squares mean (95% confidence interval)

*NA* not applicable

### Post-hoc MMRM analyses

When change from baseline differences for APOE ε4 carrier status were assessed, ε4 carriers were found to have significantly greater change from baseline relative to ε4 noncarriers (Δ = −3.15, 95% CI −5.63 to −0.66; *p* = 0.01). For progression status, progressors displayed significantly greater change from baseline than nonprogressors (Δ = −7.51, 95% CI −9.48 to −5.53; *p* < 0.001). MMRM models restricted to nonprogressors were performed to determine the effect of pathology group differences on the composite score. For high/low CERAD groups, the difference in change from baseline was not statistically significant (Δ = −1.17, 95% CI −3.39 to 1.05; *p* = 0.30). The difference for the high/low Braak groups was also not significant (Δ = 0.52, 95% CI −1.78 to 2.82; *p* = 0.66).

### *Z* score standardization of the composite score

An additional analysis was performed using a *z* score transformation to create the composite score. The individual cognitive tests at each visit were standardized to the mean and standard deviation of their baseline values, which generated a *z* score for each raw test value. The *z* scores for each individual at each visit were summed and divided by eight to create a composite *z* score. Change from baseline analyses for the composite *z* score were conducted similar to the previous MMRM analyses. The high CERAD group showed worse performance relative to the low CERAD group on the composite *z* score, but this difference was not statistically significant (Δ = −0.16, 95% CI −0.33 to 0.01; *p* = 0.06). The composite *z* score difference for the high and low Braak stage groups was also not significant (Δ = −0.03, 95% CI −0.20 to 0.14; *p* = 0.74). For the three-group analysis, the composite *z* score results were similar to the previous three-group analysis where the high CERAD/Braak group had significantly greater decline than the intermediate group (Δ = −0.33, 95% CI −0.50 to −0.15; *p* < 0.001), but not the low CERAD/Braak group (Δ = 0.00, 95% CI −0.23 to 0.22; *p* = 0.98). Similar to the previous analysis, the intermediate group had significantly better performance than the low CERAD/Braak group (Δ = 0.32, 95% CI 0.08–0.57; *p* = 0.01).

## Discussion

The results of this study demonstrate a significant association between AD-related amyloid and tau pathology and a cognitive composite score similar to those used in on-going AD prevention trials [12–14, 38, 39]. In addition, these findings serve to establish a relatively robust association between a cognitive outcome and AD pathology. Our results also provide empirical support for the use of cognitive composite scores as a primary outcome for AD prevention trials. Although many of the current AD therapies in clinical trials are focused primarily on amyloid reduction [40], the continued development of tau-directed treatments [41] will require that cognitive composite scores correlate well with both AD lesions. These results are important in light of the Food and Drug Administration’s (FDA) revised guidance for drug development in early AD [9], which indicates that approval for a new treatment could be obtained based on a “persuasive effect” on a cognitive outcome. However, the guidelines emphasize that a study sponsor must demonstrate that cognitive function is related to underlying disease pathology in a broader clinical context (certainty of diagnosis and future clinical course) [9]. Previous studies have shown that cognitive trajectories in cognitively stable individuals are impacted by the severity of AD pathology [19–21]. Here we found the same association in a mixed sample of progressors and nonprogressors. Since AD prevention trials are likely to include individuals who will progress to MCI during the course of the trial, including these subjects in our analysis provides a more accurate estimate of cognitive trajectories in the context of an AD prevention trial.

Since other currently used composite scores [13, 14, 17, 18] are comprised of the same, or similar, cognitive subtests to those employed here, it is likely that their association with AD pathology would be comparable with our composite score. Although the FDA’s revised guidance for early AD drug development [9] provides greater latitude in the use of cognitive outcomes for efficacy analyses, there is still a need to demonstrate that observed cognitive changes are associated with underlying disease pathology as suggested by the current study.

This present study also lends support to the requirement that potential subjects display positive amyloid scans to meet inclusion criteria in AD prevention trials. Since AD prevention trial inclusion criteria include clinically asymptomatic individuals with significant AD pathology who are at a higher risk for the development of cognitive symptoms, it is these individuals in which preclinical intervention(s) may significantly delay or halt the onset of clinical decline. The results derived from the CERAD high/low grouping show that individuals with high plaque load had significantly greater annualized declines on the composite score relative to those with low plaque load. In addition, the medium effect size (*d* = 0.56) indicates clinical relevance for this difference. Amyloid imaging evidence has shown that decreased performance in several different cognitive domains is associated with greater amyloid load in cortical regions associated with AD (e.g., precuneus, anterior cingulate, posterior cingulate, temporal cortex, pre-frontal cortex, etc.) [42]. Others have shown that increased plaque and NFT pathology in the entorhinal cortex, CA1, and subiculum is associated with worse antemortem memory performance [43]. Our findings are similar to these domain- and region-specific cognition and pathology associations.

MMRM analysis did not reveal significant differences in composite score trajectory between the low and high Braak groups (*p* = 0.56), indicating that the groups had similar rates of decline (Fig. 4). In addition, the MMRM analysis of the low and high CERAD groups showed that the high CERAD group had a significantly greater change from baseline relative to the low CERAD group (*p* < 0.001). Furthermore, an additional MMRM analysis revealed that individuals in the high CERAD and high Braak stage groups exhibited greater change from baseline relative to the intermediate pathology group. The lack of significance between the high CERAD/high Braak and the low CERAD/low Braak groups is surprising, but may be driven by the inclusion of eight progressors in the low CERAD/low Braak cohort that showed significant cognitive decline. Overall, these results suggest that increased plaque burden drives the observed change in the composite score, which contrasts with other studies showing that both NFTs and NPs are associated with cognitive decline [44, 45]. A study by our group found a lack of association between NFT load and cognition in a cross-sectional analysis [46], but in a subsequent longitudinal analysis we found that the interaction of higher Braak stage, older age, and positive APOE ε4 status were associated with declines in episodic memory and executive function in NCI older adults [47]. Furthermore, we previously found that decreased cognitive performance in NCI subjects in a cross-sectional study was associated with NP load and not DP load [48].

The finding that the intermediate pathology group performed significantly better than the low CERAD/low Braak stage group in the three-group MMRM analysis is curious. A possible explanation might be that cognitive reserve mechanisms allowed the intermediate group to maintain cognitive function in the presence of plaque or tangle pathology [46, 49]. However, of the 53 subjects in the intermediate group, 29 displayed high Braak pathology raising the possibility that these may be cases of neurofibrillary tangle predominant dementia (NFTPD) in which the APOE ε4 allele is less prevalent and cognitive impairment is less severe relative to sporadic AD [50, 51]. Only three of the 29 high Braak subjects were APOE ε4 carriers and this group did not show a significant decline from baseline. The lack of cognitive composite score decline in the high Braak group may also suggest the presence of primary age-related tauopathy (PART), which is characterized by the presence of NFT pathology with no or minimal amyloid plaque deposits and is associated with a lack of cognitive decline and low APOE ε4 prevalence [52]. Since these cases displayed both plaque and tangle pathology, they do not meet the criteria for PART.

Another important issue raised by this study is the relative discordance of neuropathological and clinical status and its impact on the interpretation of composite score differences. Here we found that the probable AD CERAD diagnosis and advanced Braak scores were relatively equal between progressors and nonprogressors. Heterogeneity of AD neuropathology among NCI individuals has been reported previously by our group in the population examined here [46–48], which may be related to the observation that some of the CERAD diagnostic groups were not significantly different on the annualized composite score change. When high and low pathology status was considered, annualized change was significantly lower among the high CERAD and high Braak stage groups with effect sizes that also indicated clinical relevance. Although it is unclear whether these effect sizes relate to treatment efficacy, they suggest that significant treatment effects could be observed if disease-modifying therapies are initiated early.

Composite scores have been used as clinical trial endpoints in other therapeutic areas [53], allowing for multiple components of a particular outcome to be measured in a single index. Since the clinical presentation of MCI and AD can differ in terms of which cognitive domains show the earliest decline, cognitive composite scores allow for these changes to be observed while not requiring additional statistical power if each cognitive test were treated as separate outcomes. Moreover, how components of a composite score are weighted is also an important issue [54, 55]. Donohue and colleagues [13] commented that differing weights among the components of a cognitive composite score are not easily attained since there is no a priori evidence of how a weighting scheme relates to a treatment response. In addition, implementing a fixed weighting scheme assumes a degree of homogeneity in the clinical presentation and course of decline in cognitive domains. Since decline in nonmemory domains may not occur at the same frequency or at the same trajectory between individuals, giving equal weight to the components of a composite score might allow for cognitive decline to be measured more accurately in the presence of heterogeneous clinical presentations. Others have shown relative improvement in treatment-effect detection using linear weights in a cognitive composite score [55]; however, the use of linear weighting may produce only marginal gains in statistical power [55].

Another challenge in the interpretation of the current findings is the follow-up timeframe. Although we restricted the follow-up length to 5 years to approximate that of AD prevention trials, it is reasonable to expect that many individuals may develop clinical symptoms of MCI/AD after this timeframe. Therefore, the prognostic value of these results may be limited. In this study, the composite score differences were not clear until year 4, and it is likely that these differences were driven by progressors with high pathology. An additional limitation is the relatively small number of APOE ε4 carriers, particularly homozygous individuals, which may affect the associations reported here. Future studies with a greater balance of APOE ε4 carriers and noncarriers will extend these results. The subjects in this study were from a community-based group of highly educated retired clergy who had excellent healthcare and nutrition and were used in multiple clinical pathological [56] and epidemiological investigations [35]. Individuals who volunteer may introduce bias by decreasing pathology, but this is partially mitigated by high follow-up and autopsy rates of the RROS [27]. The findings presented here may be limited to a less heterogeneous population since the individuals examined were virtually absent of vascular or other comorbid neuropathology. Since vascular lesions and other neuropathologies frequently occur in the presence of classic AD pathology, this cohort is more representative of those individuals that would be chosen for an AD clinical prevention trial.

Study strengths include uniform premortem clinical and postmortem pathological evaluation and that the final pathologic classification was performed without knowledge of the clinical evaluation. An additional strength is that our results are easily translatable to clinical trials since the MMRM approach is often used to analyze the primary outcome of AD clinical trials [57–63]. In this study, the selection of covariates is similar to that used in the efficacy analyses of clinical AD trials [57–63]. Furthermore, the cognitive composite score used in the present investigation is similar to composites currently being used in on-going AD prevention trials [12–14, 37, 38], which adds to the generalizability of our findings.

## Conclusion

The results of this study are prescient given that cognitive composite scores are being utilized as primary outcomes in AD prevention trials. The findings presented here establish that cognitive composite score performance correlates well with AD pathology in a preclinical context. By showing that a clinical outcome is associated with treatment targets of on-going AD prevention trials, these results may provide additional support for prevention trials that demonstrate beneficial treatment effects.

## Abbreviations

AD: Alzheimer’s disease
ANOVA: Analysis of variance
AR(1): Autoregressive order 1
CERAD: Consortium to Establish a Registry for Alzheimer’s Disease
CI: Confidence interval
DP: Diffuse plaque
EPAD: European Prevention of Alzheimer’s Dementia
MCI: Mild cognitive impairment
MMRM: Mixed-model repeated measure
MMSE: Mini Mental Status Examination
NCI: No cognitive impairment
NFT: Neurofibrillary tangle
NFTPD: Neurofibrillary tangle predominant dementia
NP: Neuritic plaque
PART: Primary age-related tauopathy
RROS: Rush Religious Order Study
